# Glioblastoma-on-a-chip construction and therapeutic applications

**DOI:** 10.3389/fonc.2023.1183059

**Published:** 2023-07-12

**Authors:** Zuorun Xie, Maosong Chen, Jiangfang Lian, Hongcai Wang, Jingyun Ma

**Affiliations:** The Affiliated Lihuili Hospital of Ningbo University, Ningbo, Zhejiang, China

**Keywords:** glioblastoma, organ-on-a-chip, microfluidics, bioprinting, chemotherapy, immunotherapy

## Abstract

Glioblastoma (GBM) is the most malignant type of primary intracranial tumor with a median overall survival of only 14 months, a very poor prognosis and a recurrence rate of 90%. It is difficult to reflect the complex structure and function of the GBM microenvironment *in vivo* using traditional *in vitro* models. GBM-on-a-chip platforms can integrate biological or chemical functional units of a tumor into a chip, mimicking *in vivo* functions of GBM cells. This technology has shown great potential for applications in personalized precision medicine and GBM immunotherapy. In recent years, there have been efforts to construct GBM-on-a-chip models based on microfluidics and bioprinting. A number of research teams have begun to use GBM-on-a-chip models for the investigation of GBM progression mechanisms, drug candidates, and therapeutic approaches. This review first briefly discusses the use of microfluidics and bioprinting technologies for GBM-on-a-chip construction. Second, we classify non-surgical treatments for GBM in pre-clinical research into three categories (chemotherapy, immunotherapy and other therapies) and focus on the use of GBM-on-a-chip in research for each category. Last, we demonstrate that organ-on-a-chip technology in therapeutic field is still in its initial stage and provide future perspectives for research directions in the field.

## 1 Introduction

Glioblastoma (GBM) is the most common primary malignancy of the brain, accounting for approximately 57% of all gliomas and 48% of all primary malignancies of the brain (1). It is the most aggressive glial tumor type with characteristics including a proclivity for necrosis, uncontrolled cellular proliferation, diffuse infiltration, increased angiogenesis, and widespread genomic heterogeneity (2). Despite recent advances in comprehensive treatment for GBM, including surgery, radiotherapy, and systemic therapies such as chemotherapy and targeted therapy, as well as supportive care, the overall prognosis and long-term survival rates of GBM patients remain poor (3). The most commonly used post-operative treatment regimen for GBM internationally is the ‘Stupp’ regimen, in which temozolomide (TMZ) treatment concurrent with radiotherapy is followed by TMZ adjuvant chemotherapy. A progression-free survival of 6.9 months and median overall survival of 14.6 months have been reported in newly diagnosed GBM patients treated with the Stupp regimen (4).However, there is an urgent need for more patient-specific precision therapeutic approaches for GBM to improve overall survival and quality of life of GBM patients.

Numerous studies have shown that inter-tumor and intra-tumor heterogeneity in GBM are the main reasons for unsatisfactory clinical and pre-clinical trial results (5–8). Successful targeting of GBM heterogeneity requires insight into the factors that drive sub-clonal variation, such as vascularity, hypoxia and inflammation (9). This can be achieved by advanced *in vitro* GBM models includingboth GBM tumor and normal brain tissues. However, traditional Petri-dish-based assays do not fully represent the complexity of tumors, limiting their potential use to determine predictive functional biomarkers. Organ-on-a-chip (OoC) is a revolutionary novel technology that has been developed rapidly in the past decade. Using OoC technology, human functional units constituting tissues and organs can be simulated ex vivo on microscopic cell and tissue culture vehicles, including the basic components and elements necessary for functional units, such as multicellular components, extracellular matrix (ECM) and physicochemical microenvironmental factors (10–13). OoC can compensate for the disadvantages of previous cell culture methods owing to various advantages unmatched by those of traditional methods, including three-dimensional (3D) dynamic culture, controlled physicochemical stimulation, low cost, high throughput and high reliability (14). Moreover, OoC can be used to monitor cell biology changes in real time when combined with imaging instruments, helping to better record cell behavior changes during disease states and the full range of responses to drugs. As a versatile platform, OoC can cope with challenges regarding tumor sample collection and analysis and has made considerable contributions to multiple research fields, including oncogenesis, tumor metastasis, treatment verification, drug resistance and screening, with a particularly significant role in precision oncology (15).

In recent years, OoC with microfluidics and 3D bioprinting has been used to model the GBM tumor microenvironment (TME); this technology is termed ‘GBM-on-a-chip’ (16, 17). GBM-on-a-chip can mimic the functional units of GBM tumors *in vitro*, replicating the cellular composition and anatomical structure of both the target tumor and normal brain tissues, effectively simulating *in vivo* biochemical stimuli and biophysical factors to achieve precise regulation of complex factors in the GBM TME in a spatio-temporal controllable manner (18). GBM-on-a-chip can provide bionic support at the cellular and tissue levels and has been widely used to investigate biological mechanisms and therapeutics in GBM with great potential for applications in personalized precision medicine and immunotherapy (19).

In this review, we present the microfluidics and bioprinting technologies that are currently used to construct GBM-on-a-chip models. We also review recent studies of the use of GBM-on-a-chip in a variety of treatments including chemotherapy, immunotherapy and other therapies (phototherapy, magnetic hyperthermia therapy, and focused ultrasound therapy). By describing the applications of these GBM-on-a-chip models in GBM investigation, we provide a broad perspective on the progress and future of the technology.

## 2 The GBM microenvironment and construction technologies for *in vitro* GBM-on-a-chip models

### 2.1 The GBM microenvironment

**Figure 1:**
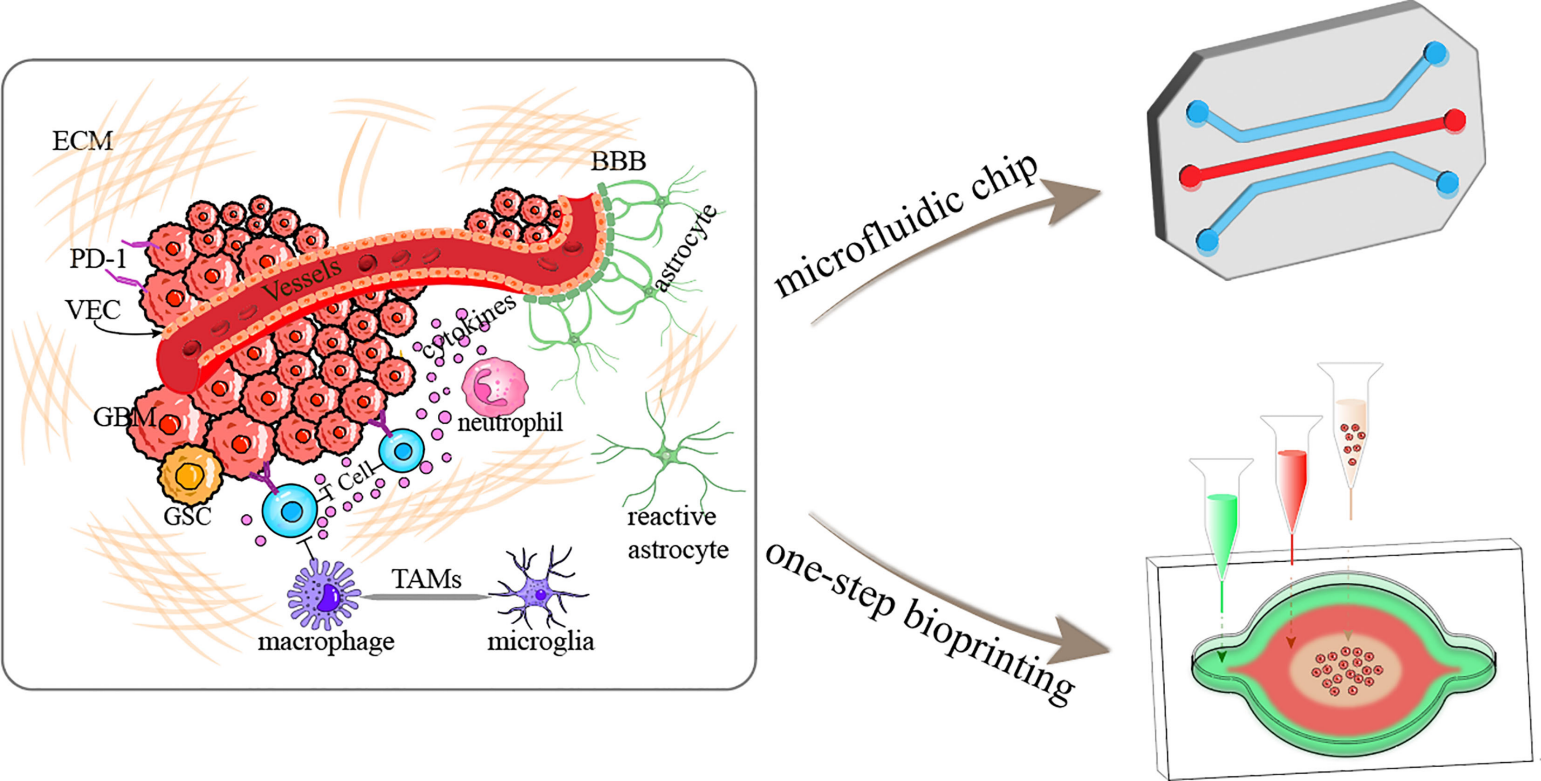
Schematic diagram illustrating the GBM tumor microenvironment and construction of GBM-on-a-chip based on microfluidics and one-step bioprinting. ECM, extracellular matrix; BBB, blood-brain barrier; PD-1, programmed cell death protein 1; VEC, vascular endothelial cell; GSC, glioma stem cell; TAMs, tumor-associated microglia and macrophages.

The TME is closely related to tumorigenesis, tumor development, and metastasis (20). In recent years, the TME has emerged as a significant participating factor and therapeutic target in GBM. The GBM TME, which refers to the sum of the internal and external environments in which GBM occurs and develops, is a complex and variable system that differs from the microenvironment in which normal brain cells and tissues are located (21). The GBM TME includes numerous cellular systems mainly represented by immune cells (tumor-associated macrophages, monocytes, and microglia (TAMs), neutrophils, regulatory T cells and bone marrow myeloid-derived suppressor cells), GBM cells, glioma stem cells, astrocytes and endothelial cells, as well as brain blood vessels, the lymphatic system, neurons, and the ECM which is essential for the microenvironment stability (22, 23) (**Figure 1**). As well as, hypoxia in the central tumor tissues, a high degree of epithelial–mesenchymal transition, high cell motility and invasive ability, disruption of the function of the blood-brain barrier (BBB), increased molecular permeability, and susceptibility to brain edema are all significant biological features of the GBM TME (24).

Overall, GBM is closely linked to the GBM TME. GBM can release cell signaling molecules to influence the TME by promoting tumor angiogenesis and inducing immune tolerance, while immune cells within the TME influence GBM cell growth and development (25). Furthermore, the non-tumor elements of the TME have a clear role in promoting GBM cell proliferation and invasion (26). The presence of the GBM TME enhances the capacity for GBM cell proliferation, migration and immune escape, thereby promoting the development of GBM. There is a relationship between the genetics of tumors and the complexity of their surrounding microenvironment. In view of the unsatisfactory results of current treatments for GBM, extensive and in-depth investigation into mechanisms of GBM development in the TME especially the relationship between the complexity of the surrounding TME and tumor genetics, is probably needed to provide new targets and new therapeutic regimens for GBM treatments (27).

### 2.2 GBM-on-a-chip models based on microfluidics

Most organ- or tumor-on-a-chip systems, such as GBM-on-a-chip models, are constructed using microfluidics technology, and the majority of microfluidic devices have been fabricated through photolithography and soft lithography (28, 29). The main polymeric material used to manufacture microfluidic chips is polydimethylsiloxane (PDMS), which offers advantages in terms of transparency, biocompatibility, flexibility, gas permeability, and resolution, giving it a dominant position in the field (30).

**Figure 2:**
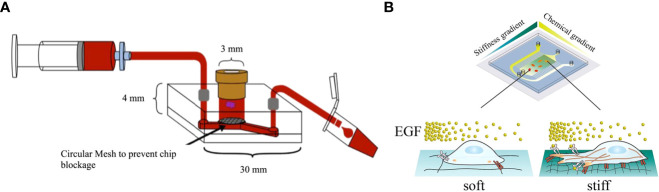
GBM-on-a-chip models constructed by microfluidic technology. **(A)** Schematic diagram of a microfluidic culture device setup (31). **(B)** Image of a microfluidic system allowing stiffness and chemical gradients simultaneously (32). Reproduced with permission from (31, 32).

Olubajo et al. used standard photolithography and wet etching techniques to fabricate a microfluidic chip featuring inlet and outlet channels (**Figure 2A**) (31). This chip was designed to cultivate 128 GBM tumor samples from 33 distinct patients in an *in vitro* fluid flow environment. The system was equipped with continuous nutrient circulation and waste removal, allowing for an average cultivation period of 72 h. The tissue viability as analyzed by flow cytometry was 61.1% in tissue maintained on the microfluidic platform after 72 h, compared with 68.9% for fresh tissue, demonstrating that patient-derived GBM tissue could be successfully maintained within the microfluidic chip to model biological processes and tissue structures of tumors for the mechanistic and therapeutic investigation in GBM. In another study, Dou et al. used soft lithography to create a polyacrylamide hydrogel-based GBM model that could precisely generate orthogonal chemical stimulation and controllable stiffness gradients to investigate the biological behaviors of GBM cells (**Figure 2B**) (32). They reported that the morphology, migration, and reactive oxygen species level of GBM cells could be regulated by increasing hydrogel stiffness, whereas the epidermal growth factor gradient could accelerate cell migration. Liu et al. developed a microfluidic device by photolithography to co-culture U87-MG cells and human umbilical vein endothelial cells (HUVECs) within a macroporous gelatin transglutaminase hydrogel to mimic a tumor-microvascular environment according to physiological conditions for studying antioxidants effects of GBM cells *in vitro* (33). Antioxidant diffusion from the HUVEC formed vessel lumen to U87-MG cells reflected the drug transportation and permeation functions of the tumor vessel.

In conclusion, microfluidics enable reproduction of the GBM TME with a reduced size chip, in particular, combination of the BBB with the tumor tissue. However, such chips usually need to be integrated with other devices as they do not have the capacity for entire laboratory operations. Moreover, the low manufacturing efficiency of PDMS-based microfluidic devices makes them unsuitable for mass production, limiting the commercialization of microfluidic systems.

### 2.3 GBM-on-a-chip models based on 3D bioprinting

As well as microfluidics, bioprinting can be used to develop refined GBM-on-a-chip models, allowing simultaneous 3D printing of specific elements such as various types of cells and ECM mimetic materials directly onto a cell-compatible substrate that can be used to form vascular networks and reproduce the heterogeneous TME (34). Furthermore, researchers can collect cells from GBM patients and construct *in vitro* tumor-on-a-chip models with biochemical and biophysical properties of GBM, which can replicate the structure of their counterparts *in vivo* and the corresponding genetics of GBM patients.

**Figure 3:**
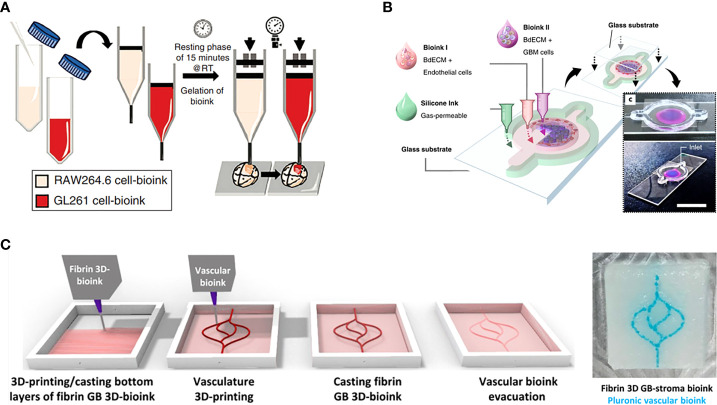
GBM-on-a-chip models constructed by one-step 3D bioprinting. **(A)** Schematic images of 3D-bioprinted mini-brain with two GelMA-gelatin bioinks containing macrophages and GBM cells (37). **(B)** 3D bioprinted GBM-on-a-chip with construction of a concentric ring structure by using various bioinks and other materials (16). **(C)** The process for 3D-bioprinting the vascularized GBM model with 3D-bioprinted vascular bioink containing GBM tumor cells and stromal cells (38). Reproduced with permission from (16, 37, 38).

In recent years, GBM models bioprinted with a unique combination of cells and bioinks have been increasingly used for further research into biological mechanisms of GBM and pre-clinical studies of GBM therapies. As an example, a GBM tumor was printed within a hydrogel system containing macrophages by extrusion-based bioprinting to build a bionic GBM TME for the investigation of the effects of infiltrating immune cells on GBM cell behavior and drug responses (35). In addition, 3D bioprinting was used to develop a novel vascularized GBM-on-a-chip model to mimic the pathophysiological conditions of GBM tumors and the surrounding vascular microenvironment, showing that gravitational force has a significant role in GBM mechanical regulation (36). Heinrich et al. constructed a 3D bioprinted GBM model to investigate the interactions between macrophages and GBM cells and for testing of drugs targeting this interaction (**Figure 3A**) (37). This GBM model was bioprinted using a bioink encapsulating RAW264.7 (a mouse macrophages cell line), and GL261 (mouse GBM cells) implanted with bioink used to fill the cavity, where the construct was subsequently photo-crosslinked.

Such 3D bioprinting can also be used to construct a patient-specific GBM-on-a-chip with patient derived GBM cells and viable bioinks to better mimic the GBM TME. For example, Yi et al. constructed a GBM-on-a-chip model based on extrusion-based bioprinting for the testing of tumor-killing drug candidates and screening of effective treatments for GBM patients resistant to standard drug therapy (**Figure 3B**) (16). In this work, patient-derived GBM cells, HUVECs, and brain-derived ECM were printed into a separated concentric ring structure of cancer stroma that could mimic the structural, biochemical, and biophysical features of the GBM tumor while maintaining a radial oxygen gradient, representing the heterogeneous ecology of GBM. In another study, Neufeld et al. used 3D bioprinting to develop a perfusable GBM model that could reproduce various *in vivo* features of GBM including growth kinetics, invasiveness, and genetic characteristics, and was used to test drug response (**Figure 3C**) (38). The heterogenic TME was reproduced using a fibrin-based GBM bioink containing patient-derived GBM cells, astrocytes and microglia, and perfusable blood vessels were simulated using a sacrificial bioink coated with brain pericytes and HUVECs. These 3D bioprinted models demonstrate the promising potential of advanced bio-manufacturing techniques in the investigation of GBM.

Compared with GBM cell lines such as U87 that have been criticized for not accurately representing the genetic and molecular characteristics of GBM in patients, GBM models bioprinted using patient-derived cells are more credible and personalized. Although they are limited by the development of applicable bioinks that satisfactorily mimic the GBM TME, these advanced biomanufacturing techniques show promise in for applications in the study of GBM. Further work is required to develop novel bioink materials and formulations for the construction of more representative GBM models based on bioprinting rather than small animal models.

## 3 GBM-on-a-chip for therapeutic applications

### 3.1 GBM-on-a-chip for the study of chemotherapy drugs

Chemotherapy, a treatment that kills tumor cells through the use of chemicals, can prolong progression-free survival and overall survival in GBM patients. GBM tumors grow rapidly and easily recur; thus aggressive and effective individualized chemotherapy would be valuable. *In vitro* GBM-on-a-chip models can assist in identifying the sensitivity of patients to specific drugs, screening different drug combinations and guiding treatment decisions.

#### 3.1.1 GBM-on-a-chip for the study of single-agent TMZ

TMZ, an alkylating agent with antitumor activity, methylates the O6 or N7 positions of guanine residues on DNA molecules and exerts cytotoxic effects through mismatch repair of methylated adducts (39, 40). It is the first-line chemotherapy drug for GBM.

**Figure 4:**
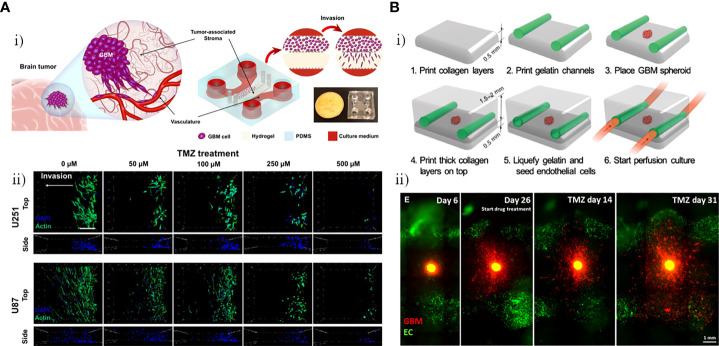
GBM-on-chips for the study of single-agent TMZ. **(A)** Investigation of the capacity of TMZ to inhibit invasion and induce programmed cell death in the GBM-on-a-chip model (41). **(i)** GBM-on-a-chip including side channels for nutrients and drugs to be delivered to cells and stroma compartments for culture of GBM cells. **(ii)** Evaluation of effects of different concentrations of TMZ on cytoskeleton of the U251 and U87 cells in the GBM-on-a-chip model. **(B)** Analysis of *in vitro* 3D-bioprinted GBM-on-a-chip model under long-term TMZ treatment (42). Schematic images of 3D-bioprinted GBM model and experimental results. **(i)** Process for 3D-bioprinting GBM model containing a GBM spheroid and vascular channels. **(ii)** Invasive behavior of patient-derived GBM cells at different stages (before TMZ treatment, day 26 when TMZ treatment was started, and 14 days and 31 days after TMZ treatment). Reproduced with permission from (41, 42).

To investigate the capacity of TMZ to inhibit invasion and induce programmed cell death, Samiei et al. created a multi-compartment microfluidic device in which U87 and U251-MG cells were cultured, including side channels for nutrients and drugs to be delivered to the cells and stroma compartments for culture of GBM cells (**Figure 4Ai**) (41). U87 and U251 GBM cells cultured in the 3D environment were significantly less sensitive to the drugs compared with those cultured in monolayer systems, and TMZ-induced autophagy and TMZ -induced apoptosis were observed. As shown in **Figure 4Aii**, there was a decrease in the invasiveness of U87 and U251 GBM cells after treatment with TMZ, and the number of invasive cells decreased with increasing TMZ dose. Ozturk et al. used extrusion-based bioprinting to construct a microfluidic platform allowing long-term culture and drug delivery with two perfused vascular channels between which a patient-derived GBM tumor spheroid was placed for monitoring and assessment of GBM cell responses to TMZ treatment (**Figure 4B**) (42). As shown in **Figure 4Bi**, a plexiglass perfusion chamber contained the 3D tissue composed of vascular channels, and a GBM spheroid was bioprinted and cultured under medium perfusion. The inner channel surface of the vascular channels was replicated by injecting HUVECs in suspension into the channels. Overgrowth of GBM cells was found to hinder the efficacy of long-term TMZ treatment, and cell metabolic activity in the GBM spheroid decreased over time with increasing TMZ dose, demonstrating that some GBM cells remain invasive after long-term TMZ treatment. As shown in **Figure 4Bii**, after treatment with TMZ for 14 days, the GBM cells had regressed and the tumor core had shrunk, however, after 31 days of TMZ treatment, the restoration of invasiveness in some GBM cells that survived the treatment strongly promoted cell drug resistance even with continuing TMZ treatment. The main focus of conventional evaluation methods is the effect of drugs on cell viability or metabolism. By contrast, Zhang et al. used the microfluidic trypsin treatment method to analyze the effect of TMZ on single-cell adhesion of U87 GBM cells, proposing that the ability to regulate cell adhesion was also a significant aspect in drug evaluation (43). According to the results, the inhibitory effect of TMZ on U87 GBM cell adhesion strength after 6 h adhesion became stronger over time, suggesting that the efficacy of TMZ is time dependent. Lactic acid was added to the culture medium to mimic the acidic TME, which was demonstrated to effectively inhibit the effects of TMZ and promote TMZ resistance of U87 GBM cells.

#### 3.1.2 GBM-on-a-chip for the study of TMZ-based combination chemotherapy

**Figure 5:**
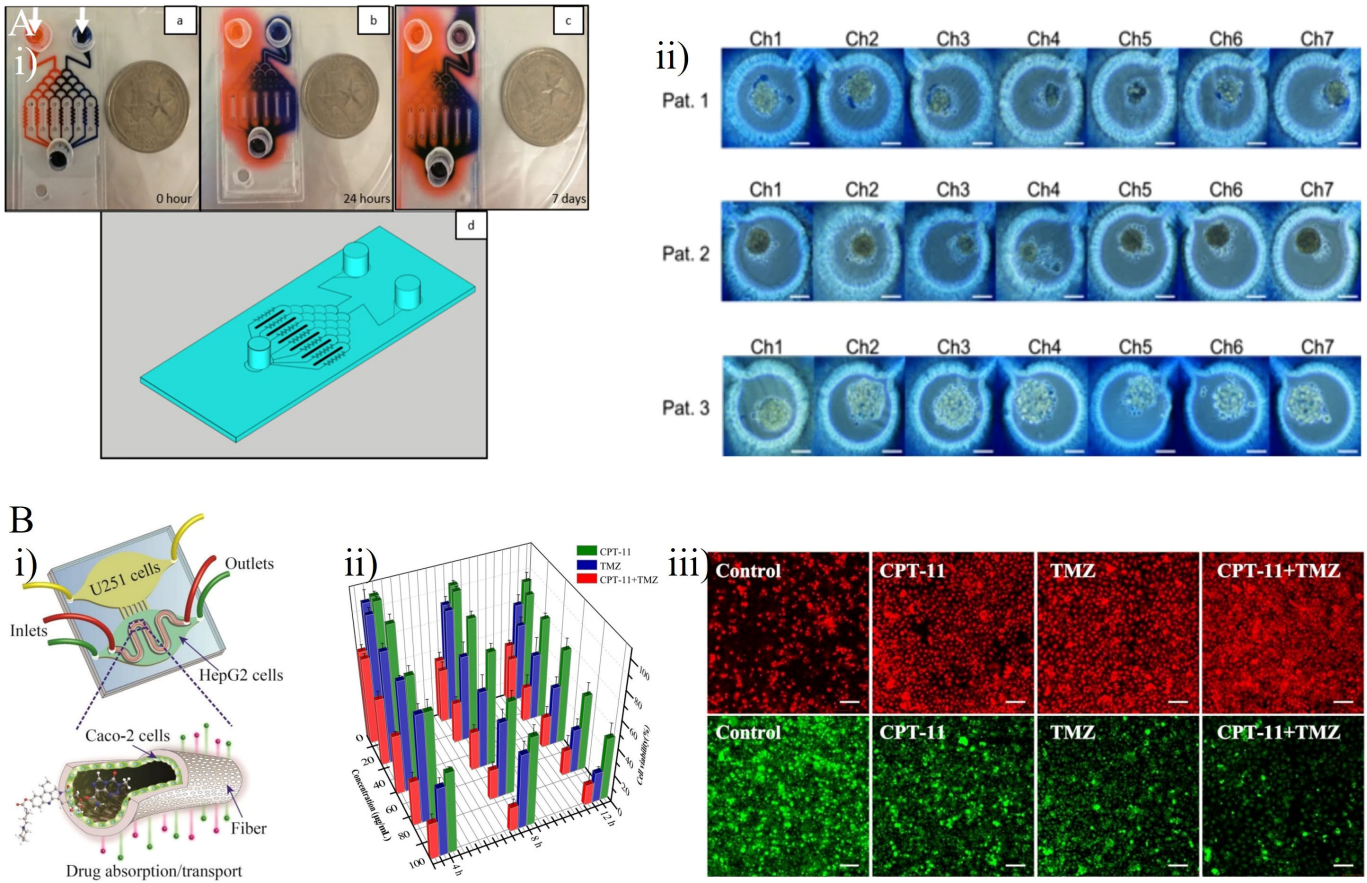
GBM-on-chips for drug studies of TMZ-based combination chemotherapy. **(A)** Patient- specific GBM-on-a-chip for testing of drug candidates (including TMZ and BEV) (44). **(i)** Two dyes were loaded into two inlets of this chip to characterize the gradients of two solutions generated in seven microfluidic channels. **(ii)** Cells loaded with 0.4% trypan blue for semi-quantitative cell viability after 7 days of drug administration. **(B)** An intestine-liver-GBM biomimetic microfluidic system for evaluating drug combination (including CPT-11, TMZ, and CP) in GBM (45). **(i)** The hollow fiber integrated microfluidic chip co-cultured Caco-2 cells, HepG2 cells, and U251 cells to simulate *in vivo* pharmacokinetic processes. **(ii)** Inhibitory effects of single- and double-drug combinations (CPT-11 and TMZ) on U251 cells after 4 h, 8 h, and 12 h treatment. **(iii)** Effects of single- and double-drug combinations on apoptosis of U251 cells. Intracellular reactive oxygen species generation (red) and glutathione reduction (green). Reproduced with permission from (44, 45).

Akay et al. constructed a GBM-on-a-chip platform with the aim of assessing the drug response of GBM cells to varying concentrations of two types of clinical anti-tumor agents, TMZ and bevacizumab (BEV) (**Figure 5A**) (44). The chip included two inlets and one outlet by which seven microfluidic channels were connected (**Figure 5Ai**). Small hydrogel-based gaps between each channel prevented the diffusion of any small drug molecules through the channel. After 7 days culture of primary human derived GBM cells from three different patients as 3D GBM spheroids, 7.5 µM BEV solution and 600µM TMZ solution were respectively applied into the GBM spheroids through the left and right channels. Single-agent TMZ was more effective than single-agent BEV as a the human GBM cell treatment, whereas TMZ in combination with BEV worked more effectively compared with mono-TMZ (**Figure 5Aii**). Ma et al. developed a detachable and assembled microfluidic device consisting of a glass cover plate coated with PDMS and a microfluidic chip constructed form PDMS, into which a multicellular spherical matrix system was integrated to mimic *in vivo* conditions. The aim was to investigate the invasive behavior of GBM cells and the anti-invasion effects of resveratrol (Res, a traditional Chinese medicine), TMZ, and the Res + TMZ combination on GBM (46). Compared with single-agent TMZ, Res in combination with TMZ treatment at the same concentration promoted the efficacy of TMZ against GBM cells, and single-agent Res also exhibited significant anti-cancer effects. These results confirmed the previous theory proposing that Res has anti-invasive and anti-proliferative effects on GBM, as well as amplifying the anti-cancer effect of TMZ against GBM (47, 48). Jie et al. developed a bionic intestine-liver-GBM system for evaluation of combination drug treatments in GBM (**Figure 5B**) (45). As effective drugs for GBM chemotherapy require the ability to penetrate the BBB and maintain pharmacological activity after metabolism in the liver, these factors have a significant role in determining the pharmacological activity of many drugs for GBM. In the microfluidic chip, Caco-2 cells were cultured in the upper layer, into which a hollow fiber was embedded to replicate an artificial intestine to deliver drugs. HepG2 cells and U251 cells were respectively cultured within two horizontally aligned olivary chambers of the bottom chamber to mimic liver metabolism and the GBM tissue (**Figure 5Bi**). After intestinal absorption and liver metabolism simulated by the intestine-liver metabolic model, Irinotecan (CPT-11), TMZ, and cyclophosphamide (CP) were applied as single- and double-drug combination therapies for GBM cells. Compared with single-drug treatments, the CPT-11 and TMZ combination showed a marked improvement in efficacy (**Figures 5Bii, iii**). When used to treat U251 cells, this combination was more effective than the CPT-11 and CP combination as well as the TMZ and CP combination.

#### 3.1.3 GBM-on-a-chip for the study of non-TMZ chemotherapy

**Figure 6:**
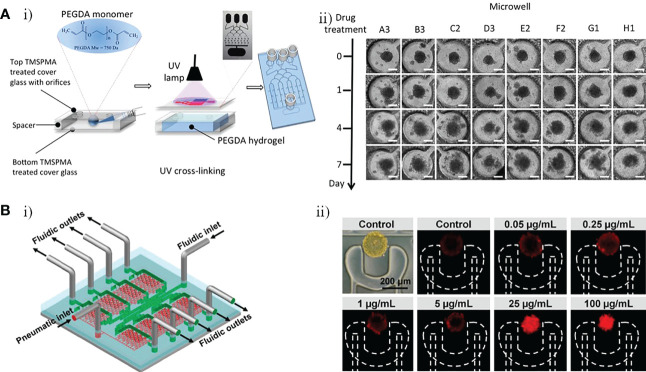
GBM-on-a-chip for drug studies of non-TMZ chemotherapy. **(A)** PEGDA hydrogel-based GBM-on-a-chip for evaluation of anti-cancer drugs (Pitavastatin and Irinotecan) (49). **(i)** Illustration of the construction of final hydrogel-based microfluidic device with microchannels and microwells. **(ii)** Images of GBM tumor spheroids in the microwells before (day 0) and after (days 1, 4, and 7) combinatorial drug treatment. **(B)** Microfluidic platform for monitoring tumor response to vincristine and bleomycin (50). **(i)** Representative schematic of a recyclable microfluidic platform. **(ii)** Effects of different concentrations of vincristine on tumor cell death after 4 days of treatment. Reproduced with permission from (49, 50).

Fan et al. developed a 3D microfluidic chip for culture of U87 GBM cells, constructed using a photopolymerizable polyethylene glycol diacrylate (PEGDA) hydrogel, to test a combination drug therapy consisting of Pitavastatin and Irinotecan (**Figure 6A**) (49). This platform with three inlets and one outlet that included a top glass cover plate, a bottom glass cover plate, and a middle layer composed of PEGDA hydrogel, could drive diffusion *via* a concentration gradient to regulate the release of chemicals. It also provided a large number of miniature culture chambers in which high-throughput GBM spheroids could be formed (**Figure 6Ai**). This enabled massive parallel testing of responses to multiple drugs with simultaneous administration in a 3D biologically compatible microenvironment. The results indicated that the Pitavastatin and Irinotecan combination worked more effectively compared with individual agent treatments, with drug efficacy measured based on the cell viability of GBM spheroids (**Figure 6Aii**). In another study, Liu et al. constructed a microfluidic device in which U251 GBM cells were cultured under various conditions to evaluate the efficacy of vincristine (VCR) and bleomycin (BLM) against GBM cells at six different concentrations (**Figure 6B**) (50). After 4 days high concentration (100 µg/mL) treatment, decreases in the tumor size and number of tumor cells were observed in both the VCR and the BLM group. Compared with BLM, VCR worked more effectively, killing more than 80% of U251 cells and reducing tumor size by 49%, whereas BLM killed about 66% of U251 cells and reduced tumor size by 30% (**Figure 6Bii**). Recently, Rahimifard et al. created a microfluidic platform to evaluate the effects of pyrazino[1,2-a] benzimidazole derivatives on patient-derived GBM cells (51). New pyrazino[1,2-a] benzimidazole derivatives were found to have obvious anti-cancer properties and COX-II inhibitory effects (52). GBM cells were exposed to subtoxic concentrations of 2,6-dimethyl pyrazino[1,2-a] benzimidazole (L1 6.5μM) and 3,4,5-trimethoxy pyrazino[1,2-a] benzimidazole (L2 42.5μM). Both L1 and L2 exhibited anti-proliferative and anti-migration properties against GBM cells, and both retarded the formation of 3D GBM spheroids.

As GBM tumors inevitably recur after surgery and radiation treatment, chemotherapy plays an important part in killing the remaining GBM cells. However, the BBB prevents the entry of adequate chemotherapeutic drugs into the cerebral circulation brain, limiting the effects of systemic chemotherapy against GBM. To overcome this limitation, there is a need for a patient-specific *in vitro* model using OoC technology that accurately represents the GBM TME, especially the BBB. Such patient-specific models could be used to screen the most appropriate drug combinations for individuals. However, owing to its lack of capacity to reflect neurotoxicity and other adverse effects on patients, the model would need to be integrated with multiple biological systems that can recapitulate the complex functionalities of different human tissues or organs so as to simulate the physiology of the patient with a high degree of fidelity. Thus, researchers could search for better chemotherapeutics to target GBM while reducing drug-induced injury.

### 3.2 GBM-on-a-chip for immunotherapeutic investigation

GBM is highly heterogeneous, and extrinsic components of tumor cells that are inherent to the brain, as well as intrinsic mechanisms of tumor cells that assist immune evasion make the GBM TME extremely challenging to cope with (53). Reducing the barrier to immunosuppression by targeting the tumor stroma may provide an opportunity to treat GBM. The immunotherapies for GBM currently be investigated using GBM-on-a-chip models can be broadly classified into immunotherapies targeting TAMs, immune checkpoint blockade (ICB) therapy, and chimeric antigen receptor T cells (CAR-T) therapy.

#### 3.2.1 Immunotherapy targeting TAMs

TAMs can secrete a variety of enzymes, reactive oxygen species, growth factors, and cytokines that contribute directly and/or indirectly to tumor proliferation, invasion and angiogenesis in GBM (54). Thus, they have an essential role in the formation of the immunosuppressive GBM TME (55, 56). Although a large number of studies have demonstrated that TAMs can promote the invasion and proliferation of GBM (57–60), the specific mechanisms by which TAMs interact with GBM cells are not known and whether they are involved in GBM recurrence and the nature of their interactions with tumor stem cells are still unclear. Therefore, in-depth study of the relationship between TAMs and GBM cells may provide the basis for immunotherapy targeting TAMs.

**Figure 7:**
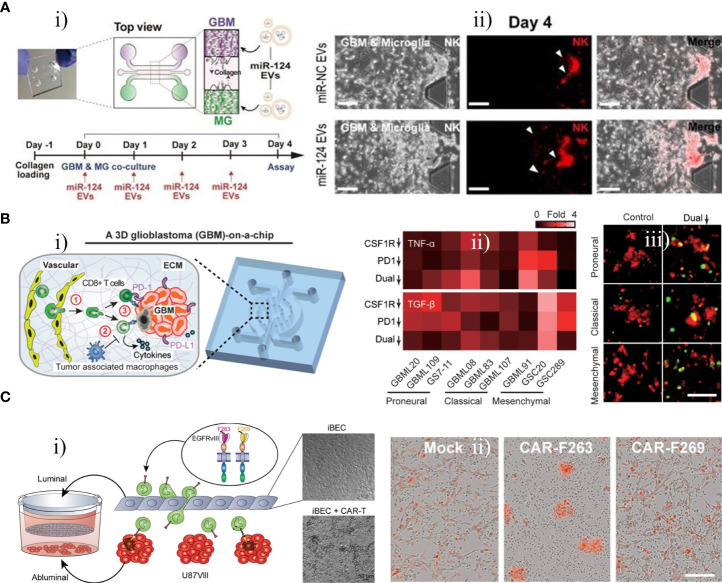
GBM-on-chips for investigation of immunotherapy. **(A)** Microfluidic co-culture device for investigating inhibition of tumor progression by miR-124 EVs (62). **(i)** Co-culture of microglia U373MG cells with miR-124 EVs for 4 days. **(ii)** On day 4, NK cells in the microfluidic device were treated with miR-negative control EVs or miR-124 EVs. **(B)** GBM- on-a-chip model of the TME for optimizing anti-PD-1 immunotherapy (63). **(i)** Schematic image of a patient-specific GBM-on-a-chip to recreate the immunosuppressive TME. **(ii)** Quantified cytokine levels showing significantly increased and decreased expression of TNF-α and TGF-β with dual inhibition therapy. **(iii)** Relative to the control group, co-blockade of PD-1 and CSF-1R resulted in more apoptotic GBM cells (green nuclei). **(C)** Transwell BBB and U87vIII co-culture model for pre-clinical evaluation of GBM-targeting CAR-T (64). **(i)** Schematic illustration of the construction of transwell BBB and U87vIII co-culture microfluidic device. **(ii)** CAR−T mediated cytotoxicity of U87vIII cells, with CAR-F263 showing a faster and stronger U87vIII killing response. Reproduced with permission from (62-64).

Gu et al. established three microfluidic assays, which they refer to as co-migration assays, based on a microfluidic device that can be used for the investigation of the bi-directional relationship between GBM cells and microglia (61). Microglia exhibited both anti-tumor and pro-tumor activities, suppressing early tumor growth by their phagocytosis and killing ability, then participating in tumor invasion and proliferation in the malignant stage to promote the tumor progression of GBM. Hong et al. developed a 3D microfluidic co-culture device to investigate the effects of microRNA (miR)-124-loaded extracellular vesicles (EVs) by recreating the interaction between microglia and GBM cells (**Figure 7A**) (62). U373-MG cells and microglia were co-cultured with miR-124 EVs for 4 days (**Figure 7Ai**). The miR-124 EVs exhibited inhibitory effects on the proliferation and metastasis of GBM and suppressed microglial M2 polarization *via* STAT3 regulation, providing initial evidence for the use of miR-124 EVs to develop a novel therapeutic strategy. The miR-124 EV treatment also suppressed tumor progression and anti-tumor immune responses, leading to enhanced intratumoral infiltration of natural killer (NK) cells (**Figure 7Aii**). Similarly, Cui et al. created a biomimetic and microfluidic-based model to mimic macrophage-associated immunosuppression and tumor angiogenesis in GBM and to investigate the antitumor function of macrophages (65). The results indicated that the regulation of tumor angiogenesis in GBM may involve TGF-β1 (soluble immunosuppressive cytokine) and surface endothelial-macrophage interactions, whereas perivascular macrophage-endothelial interactions are involved in regulating pro-angiogenic activity *via* the integrin (αvβ3). Using this GBM-on-a-chip model, a novel dual αvβ3 and TGF-β1 blockade was found to suppress tumor neovascularization of GBM by simultaneously targeting endothelial-macrophage interactions and macrophage-associated immunosuppression.

#### 3.2.2 ICB therapy

ICB therapy has achieved great success in the treatment of advanced tumors of various types, including melanoma, lymphoma, lung cancer, and kidney cancer, with significant improvements in median overall survival in recent years (66–69). Immune checkpoint inhibitors are known to promote the transition from a normal immune system to enhanced immune activation (70). The potential benefits of ICB therapy for GBM patients have attracted significant interest in recent years; however, the efficacy has been unsatisfactory to date (71).

Cui et al. developed a patient-specific GBM-on-a-chip platform for analyzing the heterogeneity of immunosuppressive TMEs and optimizing anti-programmed cell death protein 1 (PD-1, an immune checkpoint inhibitor) immunotherapy against various GBM subtypes (**Figure 7B**) (63). This platform was used to culture brain microvascular endothelial cells simulating a 3D brain microvascular environment, human macrophage-derived TAMs, molecularly distinct patient-derived GBM cells, and human primary CD8+ T cells in a biomimetic 3D brain ECM to mimic the interaction between an immune system and GBM cells (**Figure 7Bi**). Various subtypes of GBM exhibited diverse CD8+ T-cell dynamics, and a CSF-1R inhibitor could enhance the efficacy of the PD-1 inhibitor, revealing that immunotherapeutic efficacy for GBM may be improved by immune checkpoint inhibitors targeting PD-1 combined with inhibitors targeting TAM-associated CSF-1R signalling (**Figures 7Bii, iii**). This patient-specific GBM-on-a-chip platform provided a means of screening personalized immunotherapies for GBM patients. The team further designed an in silico immuno-oncology model to analyze GBM immune interactions based on patient-specific immunological characteristics and measurements of end-point data from the GBM-on-a-chip system mentioned above. This model could dynamically and comprehensively analyze the multiple mechanisms of TAM-associated immunosuppression against anti-PD-1 immunotherapy. It was further demonstrated that immune responses in GBM patients could be enhanced by co-targeting TAM-associated CSF-1R signalling and PD-1 checkpoints, especially in GBM patients who did not respond to single ICB therapy targeting PD-1 (72).

#### 3.2.3 CAR-T therapy

CAR-T therapy, a revolutionary cellular immunotherapy by which T cells are genetically modified, has been approved for specific haematological malignancies and shows potential to target a variety of solid tumors (73). EGFRvIII, a variant of the epidermal growth factor receptor (EGFR), is expressed only in tumors and represents a tumor antigen that can be targeted by CAR-T in GBM (74).

Huang et al. created a microfluidic platform based on a transwell BBB and U87vIII co-culture system for assessment of BBB extravasation of U87MG cells expressing tumor-specific mutant protein EGFRvIII (U87vIII) targeted by CAR-T (**Figure 7C**) (64). Control mock T cells, and CAR-F263 and CAR-F269 with different tonic signalling profiles (two anti-EGFRvIII-targeting CAR-T cells) were applied to the luminal side (**Figure 7Ci**). After 48 h treatment, the cell viability of the U87vIII cells decreased significantly, and activated CAR-F263 showed robust cytotoxicity against U87vIII cells. Compared with CAR-F263, CAR-F269 exhibited approximately quadruple lower efficiency in killing U87vIII cells with a similar cytotoxic profile (**Figure 7Cii**). These results demonstrate the potential of this platform in deciphering the effects of CAR-T on post-barrier target cells with concomitant toxicity and the mechanisms of BBB disruption induced by CAR-T.

In recent years, OoC technology has proved able to almost fully reproduce the GBM tumor immune microenvironment and has become a potent tool for investigation of GBM immunotherapy. However, as microfluidic chips are usually constructed using artificially engineered materials, they may not exactly replicate the real TME. Moreover, there are many geneogenous immunizing cells and adaptive immunizing cells, and the absence of one cellular component or incorrect cellular proportions may result in differences compared with the natural tumor immune microenvironment. Thus, standardization is urgently required to enable researchers to build homogenous models with standard methods that can reproduce the complexity of the GBM tumor immune microenvironment in the future.

### 3.3 GBM-on-a-chip for other therapies

#### 3.3.1 Phototherapy

Phototherapy comprises two main approaches: photodynamic therapy (PDT), and photothermal therapy (PTT). PDT can cause local chemical damage to target lesions under specific light irradiation, using a photosensitizer to produce large amounts of reactive oxygen radicals that can kill tumor cells. PTT causes local thermal damage when the photothermal agent is irradiated by light at a specific wavelength, causing it to heat up and consequently kill tumor cells (75). The use of photosensitizers is a key component of PDT, whereas there is no need for an exogenous photothermal contrast agent to increase efficiency in PTT.

**Figure 8:**
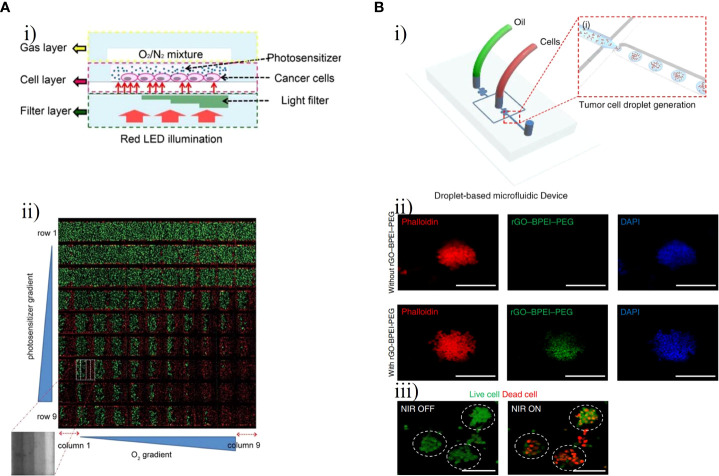
GBM-on-chips for phototherapy. **(A)** GBM-on-a-chip system for PDT screening with control of various treatment parameters (78). **(i)** Schematic illustration of the chip with control of three basic elements (photosensitizer, oxygen, and light). **(ii)** Fluorescence image of C6 cell viability after PDT treatment with horizontal channels and vertical columns. **(B)** Droplet-based GBM-on-a-chip platform for applications in PTT. **(i)** Schematic representation of the microfluidic device that generated uniformly sized 3D GBM spheroids (79). **(ii)** Fluorescent microscopic images of GBM spheroids with and without rGO-BPEI-PEG nanocomposites. **(iii)** Fluorescent microscopic images of GBM spheroids with and without NIR laser management. Reproduced with permission from (78, 79).

PDT requires three key elements, namely, a photosensitizer, oxygen, and light, to comprehensively improve its efficacy (76, 77). Lou et al. developed a microfluidic chip for high-throughput PDT assays for analysis of the efficacy of PDT against C6 cells under different treatment parameters: photosensitizer concentration, oxygen level and light level (**Figure 8A**) (78). In this chip, three layers– a gas layer, cell layer and liquid filter layer– were stacked in a glass substrate in which C6 cells were cultured and exposed to PDT under different conditions (**Figure 8Ai**). Subsequently, live/dead fluorescence staining was used to monitor cell viability, and integrated control of three key microenvironmental factors in the microfluidic system was used to comprehensively evaluate the efficacy of photosensitizer. As shown in **Figure 8Aii**, the PDT efficacy and number of dead C6 cells increased as the levels of the three factors increased. Yoon et al. synthesized methylene blue (MB)-conjugated polyacrylamide nanoparticles (PAA NPs) with a polyethylene glycol dimethacrylate (PEGDMA, Mn 550) cross-linker to improve the efficacy of PDT (80). A micro-fluidic system was developed to reliably and quantitatively measure the efficacy of PDT with MB–PEGDMA PAA NPs. The survival of C6 cells was measured with different light illumination time periods for a given MB–PEGDMA PAA NP dose; the optimal results were obtained at half maximum inhibition time under light illumination. Batches of nanoparticles were tested with three different MB loadings simultaneously on the PDT chip to determine of their cell killing efficacy.

PTT uses the light-to-heat ability of photothermal agents to enhance the heating of cells and tissues in a localized region. Cell death occurs almost instantaneously owing to protein denaturation and destruction of plasma membranes at tissue temperatures greater than 60°C, which are usually reached with PTT (81). Lee et al. constructed a photo-crosslinkable hydrogel microfluidic co-culture GBM-on-a-chip model using two-step photolithography techniques to investigate tumor metastasis and evaluate the efficacy of PTT against metastatic U87-MG cells and MCF7 human breast carcinoma cells (82). Based on the photothermal near-infrared (NIR) laser conversion function of gold nanoparticles, a new type of tumor photothermal therapy called gold nanoparticle mediated NIR thermal therapy has emerged. This has the advantages of being non-invasive and evading drug resistance and has a wide range of applications in the field of tumor thermal therapy (83, 84). After NIR laser irradiation, the viability of MCF7 and U87MG cells treated with 20 v/v% gold nanorods significantly decreased from about 90% to less than 10%, demonstrating that this treatment combination could decrease the viability of cancer cells. Lee et al. further created a droplet-based microfluidic device to evaluate the effect of PTT with a reduced graphene oxide-branched polyethyleneimine-polyethylene glycol (rGO-BPEI-PEG) nanocomposite on 3D GBM spheroids and to demonstrate the application of the 3D GBM spheroids for testing of drug response (**Figure 8B**) (79). Carbon-based nanomaterials such as rGO have unique advantages including environmental friendliness, low cost, high photothermal conversion capability, high thermal stability, and biocompatibility and are widely used in the field of photothermal devices. This microfluidic chip included two inlets for the oil phase and the aqueous phase with cultured U87-MG cells (**Figure 8Bi**). The aqueous droplets with GBM cells, the size of which could be controlled by the number of cells, were generated by a microfluidic junction producing shear forces. After 4 h of treatment with different concentrations of rGO-BPEI-PEG nanocomposites, the viability of GBM spheroids declined from 91% to 55% following NIR laser irradiation (**Figures 8Bii, iii**).

#### 3.3.2 Magnetic hyperthermia therapy

**Figure 9:**
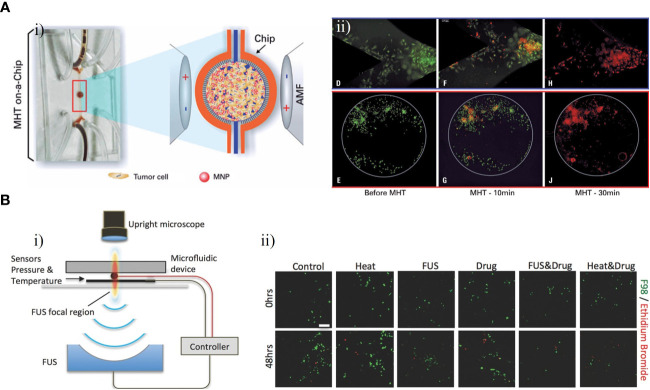
GBM-on-chips for MHT and FUS. **(A)** Microfluidic chip for evaluation of MHT (87). **(i)** In the central cavity, magnetic nanoparticles interacted with GBM cells and were then submitted an AMF. **(ii)** Viability assay for C6 cells, showing live cells before MHT and after 10 min and 30 min of MHT. **(B)** Acoustofluidic platform for controlled drug release and chemotherapy response targeting GBM (88). **(i)** Schematic illustration of the closed-loop FUS-microfluidic integrated device for drug release in GBM cells. **(ii)** Viable F98 cells and dead cells were observed before (0 h) and after (48 h) the experiment. Reproduced with permission from (87, 88).

Magnetic hyperthermia therapy (MHT) is a novel type of anti-tumor physical therapy, that takes advantage of the thermal effects of magnetic nanoparticles (MNPs) with an alternating magnetic field (AMF) and the fact that tumor cells are less heat-tolerant than normal cells. The AMF is used to selectively kill tumor cells while MNPs are injected into the tumor site (85, 86). Mamani et al. created an ‘MHT-on-a-chip’ model based on OoC technology to mimic GBM tumors, with MNPs dispersed in aqueous medium into cavities of the chip for the MHT application (**Figure 9A**) (87). The microfluidic platform included two compartments separated by a porous interface that allowed cell-to-cell interactions and cell culture in a 3D environment and microchannels allowing fluid to flow throughout the medium (**Figure 9Ai**). Through administering a flow of MNPs targeting GBM cells, this platform could mimic the dynamic TME *in vivo*. The MHT assay was performed after C6 cells had been 3D cultured in the chip for 48 h. MNPs consisting of magnetite coated with aminosilane were used to evaluate the efficacy of MHT in C6 cells. After 30 min of magnetic hyperthermia using the MNPs, nearly all GBM cells in the GBM-on-a-chip model were killed. (**Figure 9Aii**).

#### 3.3.3 Focused ultrasound

The technical principle of focused ultrasound (FUS) is to use ultrasound to penetrate human tissue without damage with a focus on the target lesion. This produces a thermal effect, force effect, and cavitation effect, resulting in direct or indirect regulation and treatment of the lesion area (89). Ultrasound delivered through the skull can be focused on a tumor for targeted ablation or used to open the BBB for delivery of drugs (90). To investigate the potential of FUS in combination with nanomedicines for treatment of GBM, Zervantonakis et al. designed a multi-layer acoustofluidic platform in which F98 rat cells were cultured in a 3D microenvironment (**Figure 9B**) (88). This platform consisted of a microfluidic chip with optical transparency and a FUS system with a closed-loop controller (**Figure 9Bi**). Temperature-sensitive liposomal carriers released DOX nanoparticles upon FUS-induced heating, resulting an increase in cellular drug uptake in the region focalized by FUS. Compared with isolated treatment groups, DOX-induced GBM cell death was increased and GBM cell proliferation in the 3D microenvironment was reduced following this treatment (**Figure 9Bii**). These results demonstrated that acoustofluidics can be used to precisely control drug release and monitor localized cell responses, and to target tumor cells regionally without causing damage to adjacent normal cells.

Phototherapy is a promising therapeutic option for cancer. To date, 5-Aminolevulinic acid-PDT has been approved by the Food and Drug Administration (FDA) for GBM treatment and has shown promising outcomes. However, its effectiveness is limited by the ability of the NIR laser to penetrate into deep brain regions. Therefore, future research should focus on increasing penetration depth in order to enhance the applicability of phototherapy. A number of challenges still need to be overcome before MHT can be applied to GBM clinical treatment, although there have been enormous advances in MHT research over the decades. For example, owing to a lack of specificity, MNPs could accumulate in healthy tissues as well as at the GBM tumor site, which might cause damage to surrounding structures. Moreover, MHT may not completely ablate the GBM tumor, leading to tumor recurrence. More research is also needed to provide sufficient clinical data to support its effectiveness and safety. A combination of phototherapy, MHT, and immunotherapy with an all-in-one microfluidic platform might be an option to achieve synergistic effects. Combined with FUS, drug-loaded microbubbles can temporally increase the permeability of the BBB and can be released at specific locations, enabling targeted delivery into the brain. However, there could be a sterile inflammatory response when the BBB is opened by FUS. In the future, emphasis should be placed on control of ultrasound parameters and the optimization of microbubble types and injection doses to achieve efficient drug delivery.

## 4 Conclusions and future perspectives

Bionic characteristics of the GBM TME, including cell-to-cell and cell-to-ECM interactions, capillaries, the BBB, and oxygen concentration gradients, can be reproduced by component construction and 3D cell arrangement with microfluidics and bioprinting in GBM-on-a-chip models. These models have considerable potential applications in studies of chemotherapy, immunotherapy, and other GBM therapies. GBM-on-a-chip models have been used to study the interactions between GBM cells and the brain microenvironment, demonstrating that GBM cells can alter the behavior of other cells in the brain, whereas the microenvironment can also influence the behavior of GBM cells. GBM-on-a-chip models have also been used to test the effects of different drugs and treatments on GBM cells in a more realistic microenvironment than those provided by traditional cell culture models. The efficacy of different drug delivery methods, such as nanoparticles and liposomes, can also be tested using GBM-on-a-chip models. However, the development of OoC technology in therapeutic fields is still in its initial stage. At present, GBM-on-a-chip models may not fully replicate the complex interactions between different cell types and the ECM that occur in the brain. Moreover, the models may not be able to capture the high heterogeneity of GBM that can vary greatly in terms of its genetic makeup and response to treatment, which could limit their usefulness in developing personalized treatment strategies for GBM patients. In the future, there is a need to build on the breakthrough of GBM-on-a-chip technology and develop more complex and bionic humanized GBM-on-a-chip models with more complex structure and function. There are still many new technologies in electrical and optical disciplines that can potentially be combined with GBM-on-a-chip, which would broaden the technical field of GBM therapy. For instance, optical-based bioprinting techniques enable rapid construction of GBM-on-a-chip models with continuous automated production. Combined with nanotechnology, GBM-on-a-chip platforms have the potential to regulate nanodrug delivery in response to electrical stimulation to facilitate targeted therapies, PPT, and PDT. Recently, the FDA has removed the requirement for animal testing prior to human clinical trials. This could represent an opportunity for OoC technology to usher in rapid development and replace animal models. One of the main advantages of OoC technology is that it can provide more accurate results than animal models. For example, a personalized GBM-on-a-chip platform can be used to develop patient-specific precision strategies and identify the best drug combination to optimize treatment outcomes in the broader GBM patient population. In the future, researchers could integrate GBM-on-a-chip with multi-organ chips to model the intersection of different biological systems. This could recapitulate organ-level physiology and pathophysiology of GBM patient, and leveraging computational modelling in combination with experimental data generated using this platform could lead to the development of effective new drugs with low side-effects and the discovery of novel therapeutic targets in GBM. As the technology continues to improve and become more widely adopted, it has the potential to transform the field of drug development and toxicology testing, while also reducing the need for animal testing.

**Table 1** summarizes the therapeutic approaches, targets, cell sources, main materials and technologies of the GBM-on-a-chip models for therapy applications that are discussed in this review.

**Table 1:** Examples of glioblastoma-on-a-chip models for therapeutic applications.

Therapeutic Approaches	Targets	Cell Sources	Main Materials	Technologies	Ref.
Chemotherapy	TMZ	U87-MG, U251-MG	PDMS	Microfluidic chip and soft lithography	(41)
Chemotherapy	TMZ	HUVECs, Patient’s primary GBM	Collagen hydrogel precursor and Gelatin from porcine skin	Three dimensional bioprinting	(42)
Chemotherapy	TMZ	U87-MG	PDMS	Microfluidic chip and soft lithography	(43)
Chemotherapy	TMZ and BEV	Patient’s primary GBM	PEGDA hydrogel	Microfluidic chip and photolithography	(44)
Chemotherapy	TMZ and Res	U87-MG	PDMS	Microfluidic chip and soft lithography	(46)
Chemotherapy	TMZ, CP and CPT-11	Caco-2, HepG2, U251-MG	PDMS and hollow fiber	Microfluidic chip and soft lithography	(45)
Chemotherapy	Pitavastatin and Irinotecan	U87-MG	PEGDA hydrogel	Microfluidic chip and soft lithography	(49)
Chemotherapy	Vincristine and Bleomycin	U251-MG	PDMS	Microfluidic chip and soft lithography	(50)
Chemotherapy	Pyrazino[1,2-a] benzimidazole derivatives	Patient’s primary GBM	PDMS	Microfluidic chip and soft lithography	(51)
Immunotherapy	GBM and Microglia	microglial BV-2 cells, C6	PDMS	Microfluidic chip and soft lithography	(61)
Immunotherapy	GBM and Microglia	U373-MG, U87-MG, Patient’s primary GBM and microglia, NK-92	PDMS	Microfluidic chip and soft lithography	(62)
Immunotherapy	Macrophage antitumor	GL261, CT-2A, RAW264.7, ATCC	PDMS	Microfluidic chip and soft lithography	(65)
Immunotherapy	anti-PD-1 immunotherapy	Patient’s primary GBM, hBMVECs, TAM, CD8+T-cell	PDMS	Microfluidic chip and soft lithography	(63)
Immunotherapy	anti-PD-1 immunotherapy	Patient’s primary GBM, hBMVECs, TAM, CD8+T-cell	PDMS	Microfluidic chip and soft lithography	(72)
Immunotherapy	CAR-T	U87-MG Human’s primary T cells	Collagen and Fibronectin	NR	(64)
Photodynamic therapy (PDT)	PDT by MB combined with hypoxic conditions	C6	PDMS	Microfluidic chip and soft lithography	(78)
PDT	PDT by MB-PEGDMA PAA NPs	C6	PDMS	Microfluidic chip and soft lithography	(80)
Photothermal therapy (PTT)	PTT by gold nanorods	U87-MG, MCF7	PDMS	Microfluidic chip and soft lithography	(82)
PTT	PTT by rGO-BPEI-PEG	U87-MG	PDMS	Microfluidic chip and soft lithography	(79)
Magnetic hyperthermia therapy (MHT)	MHT by iron oxide coated with aminosilane	C6	PDMS	Microfluidic chip and soft lithography	(87)
Focused ultrasound (FUS)	FUS and doxorubicin-TS-liposomes	F98-GFP, Bend3	PDMS	Microfluidic chip and soft lithography	(88)

TMZ, temozolomide; U87-MG and U251-MG, two types of glioblastoma cell lines; PDMS, Polydimethylsiloxane; HUVECs, human umbilical vein endothelial cells; BEV, bevacizumab; PEGDA, poly(ethylene) glycol diacrylate; Res, Resveratrol; CP, cisplatin; CPT-11, irinotecan; HepG2, liver hepatocellular carcinoma cell line; Caco-2, colorectal adenocarcinoma cell line; C6, rat glioblastoma cell line; U373-MG, glioblastoma astrocytoma cell line; GL261, CT-2A, mouse glioblastoma cell lines; hBMVECs, human brain microvascular endothelial cells; RAW264.7, ATCC, mouse macrophage cells; CAR-T, chimeric antigen receptor T-cell; MB–PEGDMA PAA NPs, methylene blue conjugated polyacrylamide nanoparticles with a polyethylene glycol dimethacrylate cross-linker; MCF7, breast cancer cell line; rGO-BPEI-PEG, reduced graphene oxide-branched polyethyleneimine-polyethylene glycol; TS, temperature-sensitive; F98-GEP, glioblastoma cell line; Bend3, endothelial cell line of mouse brain.

## Author contributions

Conceptualization, JM and ZX; writing—original draft preparation, ZX; writing—review and editing, JM and HW; supervision, MC, and JF. All authors contributed to the article and approved the submitted version.
